# The Role of the Inflammatory Prognostic Index in Patients with Non-ST Elevation Myocardial Infarction Undergoing Percutaneous Coronary Intervention

**DOI:** 10.3390/jcm14134491

**Published:** 2025-06-25

**Authors:** Ersan Oflar, Muhsin Kalyoncuoğlu, Atilla Koyuncu, Cennet Yıldız Erbaş, Hasan Ali Sinoplu, Fahrettin Katkat, Gündüz Durmuş

**Affiliations:** 1 Department of Cardiology, Bakırköy Sadi Konuk Training and Research Hospital, University of Health Sciences, 34147 Istanbul, Turkey; ersanoflar@hotmail.com (E.O.); atikoyuncu@gmail.com (A.K.); cennet_yildiz@live.com (C.Y.E.); hasanalisinoplu@gmail.com (H.A.S.); 2 Department of Cardiology, Istanbul Training and Research Hospital, University of Health Sciences, 34098 Istanbul, Turkey; fahrettin_katkat@hotmail.com; 3 Department Cardiology, Dr. Siyami Ersek Thoracic and Cardiovascular Surgery Education Research Hospital, University of Health Sciences, 34668 Istanbul, Turkey; drgunduzdurmus@gmail.com

**Keywords:** C-reactive protein, serum albumin, neutrophil, lymphocyte, inflammatory prognostic index, myocardial infarction, prognosis

## Abstract

**Background/Objectives:** To evaluate the prognostic role of the inflammatory prognostic index (IPI) value at admission in major adverse cardiovascular and cerebrovascular events (MACCEs) in individuals with non-ST elevation myocardial infarction (NSTEMI) undergoing percutaneous coronary intervention (PCI). **Methods:** A total of 1142 NSTEMI patients with a mean age of 61.9 ± 12.5 years were included. Admission C-reactive protein level, serum albumin level, and complete blood counts of participants were collected from hospital records. The IPI was calculated based on the following formula: C-reactive protein/albumin ratio (CAR) x neutrophil-to-lymphocyte ratio (NLR). An aggregate index of systemic inflammation (AISI) value was calculated using the ‘‘neutrophil count x monocyte count x platelet/lymphocyte count’’ formula. The study cohort was divided into two groups according to the median IPI value. **Results:** Patients with higher IPI values were statistically more likely to suffer from MACCEs within one year (*p* < 0.001), thus the admission IPI value was found to be associated with future development of MACCEs. Furthermore, it had sufficient discrimination power (AUC = 0.70) and predictive accuracy in identifying MACCEs compared to other inflammatory parameters such as the CAR (AUC = 0.64), the NLR (AUC = 0.64), and the AISI (AUC = 0.59). Adding the IPI to the baseline multivariable logistic regression model significantly improved the model’s discrimination and net clinical benefit effect for identifying patients who would suffer from MACCEs, with a C-index of 0.84 (95% CI: 0.82–0.86) and explanatory power of 23.2% (R^2^ = 0.232, DeLong test *p* = 0.001). High-risk patients with an IPI value greater than 2.43 had significantly more adverse events (*p* < 0.001). **Conclusions:** The IPI may be a promising inflammatory index for use in clinical practice to determine the risk prediction of MACCEs in NSTEMI patients undergoing PCI.

## 1. Introduction

Among the various forms of acute coronary syndrome (ACS), non-ST elevation myocardial infarction (NSTEMI) occurs most frequently, representing nearly 75% of all diagnoses. Despite medical advances, it continues to be a primary driver of illness and death across the globe [1,2,3]. While patients with NSTEMI generally have a better short-term outcome compared to those with ST elevation myocardial infarction (STEMI), their long-term prognosis remains poor despite advancements in medical technology [3,4,5]. Thus, risk stratification of NSTEMI patients by identifying modifiable clinical and/or biochemical risk factors, along with thorough longitudinal management of patients with NSTEMI, is extremely important to improve overall prognosis [6]. Indeed, it has been suggested that the use of biomarkers in early risk stratification may be helpful in estimating clinical outcomes in individuals with acute coronary syndrome [7,8].

Systemic inflammation plays a key role not just in the onset and worsening of atherosclerosis, but also in triggering acute coronary thrombosis and increasing the risk of future cardiac complications [9,10]. In fact, several studies have indicated that elevated inflammatory activity and the activation of atherosclerotic plaques are central mechanisms contributing to clinical instability and adverse outcomes in patients with NSTEMI, beyond being a causative factor for the disease [10,11]. Indeed, several research studies published in recent years have focused on the prognostic-determining and predictive roles of various inflammatory biomarkers, including markers like the neutrophil-to-lymphocyte ratio (NLR), aggregate index of systemic inflammation (AISI), C-reactive protein-to-albumin ratio (CAR), and the inflammation prognostic index (IPI), in which were demonstrated the close relationship between such indices and adverse events and poor outcomes in ACS patients [1,12,13,14,15].

The IPI, a newly defined indicator of systemic inflammation, has been linked to several unfavorable outcomes in cardiovascular disease (CVD), including the no-reflow phenomenon, a higher likelihood of myocardial infarction (MI), first-time occurrence of atrial fibrillation, and contrast-induced nephropathy [14,16,17]. Furthermore, it has been suggested that the IPI demonstrates superior predictive performance compared to the CAR and the NLR, which have previously been associated with poor cardiovascular outcomes [14]. However, as far as the existing evidence suggests, there is currently a lack of research evaluating the IPI as a prognostic tool in NSTEMI populations undergoing percutaneous coronary intervention (PCI).

At present, assessing risk in NSTEMI patients primarily relies on clinical evaluation, ECG findings, angiographic results, and biomarkers indicating cardiac injury. However, well-recognized risk assessment tools, including the thrombolysis in myocardial infarction (TIMI) and the GRACE score, do not include inflammatory markers. On the other hand, there are data suggesting that the inclusion of inflammatory indices may increase the prognostic value of these validated risk scores [10]. From this perspective, we aimed to investigate the prognostic role of the IPI in the long-term follow-up of NSTEMI patients in comparison with the CAR, NLR, and AISI, which have previously been shown to contribute to outcome prediction in this population. Additionally, another objective was to determine whether the combination of inflammatory biomarkers provides a significant improvement in cardiovascular risk prediction beyond traditional risk factors.

## 2. Materials and Methods

### 2.1. Study Population

**Figure 1:**
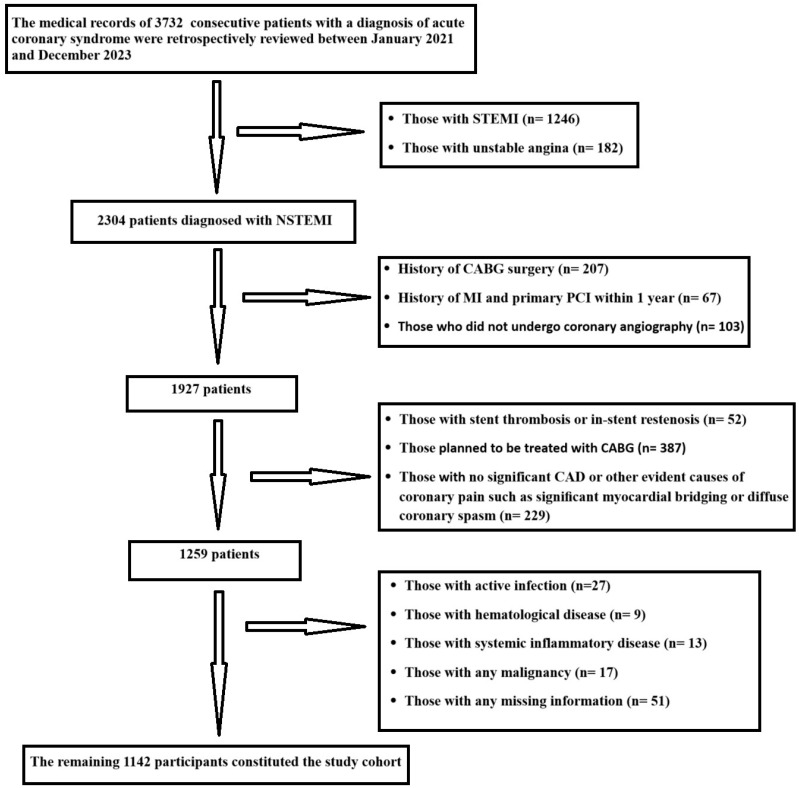
Flow chart of participant selection process and exclusion criteria.

We retrospectively analyzed the medical records of consecutive patients admitted with a diagnosis of ACS to the Department of Cardiology at Bakırköy Dr. Sadi Konuk Training and Research Hospital, affiliated with the University of Health Sciences in Turkey, from January 2021 to December 2023. Data compiled from the database of NSTEMI patients who underwent coronary angiography followed by PCI during the index hospitalization as part of standard clinical care were examined. The records of 3732 patients diagnosed with ACS in our database were retrospectively reviewed and analyzed. After excluding 1246 patients diagnosed with STEMI and 182 patients diagnosed with UA, the data of the remaining 2304 NSTEMI patients were examined. Exclusion criteria of the study were as follows: a history of coronary artery bypass grafting (CABG), a history of MI and primary PCI within the last year, no coronary angiography, the presence of stent thrombosis or stent restenosis, CABG planned after coronary angiography, the absence of significant coronary artery stenosis, the presence of other possible causes such as significant myocardial bridging or diffuse coronary spasm that could cause coronary pain, active infection, hematological disease, systemic inflammatory disease, any malignancy, and any missing data. Following the application of predefined inclusion and exclusion criteria, a total of 1142 eligible patients were included in the final analysis. A detailed overview of the selection process and reasons for exclusion is illustrated in the flow diagram (Figure 1).

Since our study was designed as a retrospective study, written consent could not be obtained from the participants, but our study protocol was approved by the Ethics Committee of the Health Sciences University Bakırköy Dr. Sadi Konuk Training and Research Hospital TUEK on 19 March 2025 (Approval number: E-2025-06-28).

### 2.2. Demographic and Clinical Characteristics

The clinical and medical histories of the patients were obtained by reviewing their electronic health records. These included personal details like BMI and family medical history; lifestyle factors such as current smoking habits; ongoing health conditions including high blood pressure, diabetes, and cholesterol issues; as well as any history of heart problems (previous heart attacks, coronary issues, stent placements, or circulation problems in the limbs). We also noted any past chronic heart failure and kidney disease, including whether patients required dialysis treatment.

Patients were considered hypertensive if their blood pressure readings during clinical visits showed systolic pressure reaching or exceeding 140 mmHg, or diastolic pressure at or above 90 mmHg, with these elevated readings documented on at least two separate occasions [18]. Diabetes was identified in patients who had a fasting blood sugar level greater than 126 mg/dL or a random blood glucose measurement exceeding 200 mg/dL [19]. Hyperlipidemia was defined as a fasting total cholesterol concentration of ≥200 mg/dL and/or a low-density lipoprotein cholesterol level of ≥130 mg/dL, according to the National Cholesterol Education Program III recommendations [20]. Active smoking status was defined as smoking more than 10 cigarettes per day for at least one year and having not made any effort to stop smoking. Family history was considered positive when heart disease or sudden cardiac death had occurred in a close male relative (parent, sibling, or child) before age 55, or in a close female relative before age 65. Peripheral arterial disease (PAD) was defined as evidence of atherosclerotic disease in blood vessels other than the coronary arteries and aorta. This diagnosis was based on matching symptoms and physical exam findings, records of previous vascular procedures to restore blood flow, history of amputation, or angiography results showing more than 50% narrowing of affected arteries [21]. Chronic heart failure was defined as a documented history of heart failure symptoms and objective evidence of cardiac dysfunction or a left ventricular ejection fraction of <40%. Chronic renal failure (CRF) was defined as the presence of kidney damage (albuminuria of at least 30 mg/24 h, hematuria, or structural abnormalities such as polycystic or dysplastic kidneys) or an estimated glomerular filtration rate (eGFR) of less than 60 mL/min/1.73 m^2^, regardless of cause, persisting for three months or longer [22]. Additionally, results from the clinical assessment of all participants were obtained as part of the routine evaluation.

The diagnosis of NSTEMI was made based on symptoms, electrocardiographic findings, and cardiac enzymes in accordance with the European Society of Cardiology and the American College of Cardiology/American Heart Association guidelines [23,24]. In addition, treatment of all patients was conducted in accordance with established protocols. The clinical manifestation of acute chest pain or severe dyspnea without persistent ST elevation on ECG is indicative of NSTE-ACS (excluding cases of true posterior myocardial infarction). NSTE-ACS subtypes were differentiated by using cardiac biomarkers of necrosis, particularly troponin. NSTEMI was confirmed when cardiac biomarkers exceeded normal thresholds alongside an appropriate clinical presentation, and individuals without biomarker elevation were diagnosed with unstable angina pectoris [23,24]. The Global Registry for Acute Coronary Events (GRACE) score was used to determine each patient’s risk level, and we included individuals from all GRACE score-determined risk groups in the current study [25]. Hemodynamic instability was defined as the presence of hypotension (systolic blood pressure below 90 mmHg or mean arterial pressure below 70 mmHg), tachycardia (heart rate of 100 bpm or higher), and signs of inadequate vital organ perfusion, such as reduced urine output and mental status changes [26].

### 2.3. Laboratory Parameters

Admission venous blood samples taken from the antecubital vein were used for routine complete blood count (CBC) testing, including neutrophil, lymphocyte, monocyte, and platelet levels, and for analysis of biochemical parameters, including blood C-reactive protein and serum albumin. Blood parameters were determined by the clinical laboratories of Bakırköy Dr. Sadi Konuk Training and Research Hospital using an automatic blood cell counter (Coulter LH 780 Hematology Analyzer, Beckman Coulter, Inc., Galway, Ireland) and an automatic chemistry analyzer (Siemens Healthcare Diagnostic Products kits and calibrators, Marburg, Germany).

Inflammation-based biomarkers were measured by using the same blood samples collected when patients were admitted. The CAR was determined by dividing the CRP level (mg/dL) by the serum albumin concentration (g/dL). The NLR was calculated as the ratio of neutrophil count (10^9^/L) to lymphocyte count (10^9^/L). The IPI was calculated according to the patient’s CAR and NLR, as IPI = CAR × NLR. The AISI was calculated by multiplying the neutrophil, monocyte, and platelet counts together, then dividing this product by the lymphocyte count, expressed as the AISI = neutrophil count (10^9^/L) × monocyte count (10^9^/L) × platelet count (10^9^/L)/lymphocyte count (10^9^/L). The study population was divided into two groups according to the median IPI values of the patients: those with low IPI and those with high IPI values.

Left ventricular ejection fraction (LVEF) was measured using the modified Simpson method in the apical four- and two-chamber views in both end-diastole and end-systole.

### 2.4. Coronary Angiography Findings

All coronary angiograms were digitally recorded using a DICOM viewer (MedCom GmbH, Darmstadt, Germany) to perform quantitative analysis. Coronary angiographies were analyzed by two interventional cardiologists who were blinded to the patients’ clinical history, laboratory results, and demographic data. The anatomic severity of coronary stenosis was quantitatively assessed by calculating the anatomical syntax score (SxSI), using the most recent version of the scoring system that we downloaded from the official website, www.syntaxscore.com (accessed on 1 January 2021).

### 2.5. Study Endpoint

The primary endpoint of our study was Major Cardiovascular and Cerebrovascular Adverse Events (MACCEs) based on the Academic Research Consortium-2 consensus. These MACCEs included cardiovascular death (resulting from myocardial infarction, major cardiac arrhythmia, heart failure, or any stroke), non-fatal myocardial infarction, or non-fatal cerebrovascular events (either ischemic stroke or transient ischemic attack) occurring within the 12-month follow-up period [27]. Information on MACCEs was collected from multiple sources, including the national death notification system, the hospital medical records database, and direct follow-up interviews with patients in person or by telephone.

### 2.6. Statistical Analysis

Normally distributed continuous variables are expressed as means ± standard deviations, while those not normally distributed are reported as medians with interquartile ranges. Categorical variables are reported as percentages, and the chi-square (χ^2^) test was used to compare variables between groups. Whether the variables were normally distributed was evaluated using the Kolmogorov–Smirnov test. A Student’s *t*-test or the Mann–Whitney U test was used to compare continuous variables between groups, depending on whether they showed normal distribution.

Correlation coefficients were calculated to determine the relationships between parameters. While the Pearson correlation coefficient was used for normally distributed parameters, the Spearman correlation coefficient was used for pairs in which at least one parameter did not exhibit a normal distribution. Correlation was defined as good when the coefficient exceeded 0.3 and as poor when it was below 0.3 [28].

To identify independent predictors of MACCEs, variables showing significance in univariate analysis were included in a multivariate Cox regression analysis, and the results were reported as hazard ratios (HRs) with 95% confidence intervals (CIs). To avoid model overfitting, an ‘event per variable’ ratio of at least 10 was maintained in the multivariable model, and only variables with *p*-values below 0.05 were included in the univariable analysis. Furthermore, the variance inflation factor (VIF) was calculated using a cut-off value of less than 5 to address possible multicollinearity among the independent variables. The predictive accuracy and discriminatory capacity of the IPI, NLR, CAR, and AISI for determining MACCEs were also calculated using receiver operating characteristic (ROC) curves and area under the ROC curves (AUC) with 95% confidence intervals. Discriminatory power was defined as ‘good’ when the AUC was 0.70 or higher and ‘inadequate’ when it was below 0.70 [29]. In addition, risk prediction model performance was evaluated using multiple discrimination and calibration metrics at 365 days follow-up. Three sequential models were developed: (1) the base model, (2) the base + GRACE model, and (3) the base + GRACE + IPI model. Harrell’s C-index, with 95% confidence intervals, was calculated for each model. Model improvement was assessed using Net Reclassification Improvement (NRI) and Integrated Discrimination Improvement (IDI) compared to the base model. The Nagelkerke R^2^ was calculated to assess the models’ explanatory power. The DeLong test was used for AUC comparisons among models [30]. The optimal IPI cut-off value was also determined by calculating the maximum sensitivity and specificity point using Youden’s index [31].

Time-to-event data were presented using Kaplan–Meier survival curves and log-rank tests. Statistical significance was set at *p* < 0.05. All statistical analyses were performed using SPSS version 24.0 (IBM Corp., Armonk, NY, USA) and R program version 3.6.3. (R statistical software, Institute for Statistics and Mathematics, Vienna, Austria).

## 3. Results

### 3.1. Baseline Characteristics

The study cohort comprised 1142 individuals with NSTEMI undergoing PCI with a mean age of 61.9 ± 12.5 years. There were statistically significant differences between individuals with low and high IPI values in terms of age; gender; history of DM, HT, HF, and CRF; presence of high Killip class; hemodynamic instability; and GRACE score. Individuals with higher IPI values were statistically more likely to experience both 30-day and 1-year MACCEs. Differences between the groups in terms of demographic and clinical characteristics are summarized in detail in Table 1.

Considering the biochemical and hematological parameters, we found that patients with a high IPI value had lower eGFR (66.5 ± 24.6 mL/min/1.73 m^2^ vs. 83.8 ± 22.4 mL/min/1.73 m^2^), lower serum albumin (3.73 ± 0.50 g/dL vs. 3.91 ± 0.47 g/dL, *p* < 0.001), higher CRP (median 7.76 mg/dL vs. median 4.95 mg/dL, *p* < 0.001), higher baseline troponin I (median 0.07 ng/mL vs. median 0.06 ng/mL, *p* = 0.047), higher neutrophil counts (5.76 ± 2.0 × 10^9^/L vs. 5.13 ± 1.8 × 10^9^/L, *p* < 0.001), fewer lymphocyte counts (median 1.74 × 10^9^/L vs. median 2.13 × 10^9^/L, *p* < 0.001), and higher monocyte counts (median 0.57 × 10^9^/L vs. median 0.53 × 10^9^/L, *p* = 0.037). In terms of the inflammation-based scores, the high IPI group had higher NLR (median 3.05 vs. 2.29, *p* < 0.001), higher CAR (median 2.0 vs. 1.28, *p* < 0.001), higher AISI (median 429.9 vs. 289.9, *p* < 0.001), and higher IPI (median 6.08 vs. 2.99, *p* < 0.001) values. Table 2 contains a comprehensive compilation of laboratory findings for the study population.

### 3.2. Independent Predictors of MACCE Development

Throughout our mean follow-up duration of 338 ± 81 days, MACCE was observed in 148 (13.0%) patients, and CV-caused death occurred in 51 (4.5%) patients. Among the patients who developed MACCEs, 90 (7.9%) patients experienced a nonfatal MI, and 7 (0.6%) patients had a stroke.

We performed multivariable Cox regression analysis to identify independent predictors of MACCEs, incorporating all variables that demonstrated significant association with MACCEs in univariate analysis, with the complete results presented in Table 3. First of all, no variable that is a member of the IPI, such as the CRP, albumin, the CAR, and the NLR, could be included in the multivariate analysis because they could negatively affect the test results. In addition, the AISI was not included in the models because it could negatively affect the results, as the IPI and AISI share some variables. Moreover, among the parameters constituting the GRACE score, CHF and troponin were included in the regression analysis because they showed a poor correlation with the GRACE score (r = 0.176 and r = 0.079, respectively). Meanwhile, other parameters related to the GRACE score, such as age (r = 0.661), CRF (r = 0.327), eGFR (r = 0.541), and Killip class (r = 0.320), were not used in the model table due to the fact that they had good correlation with the GRACE score. So, we ran three sequential models: (1) base model including gender, CAD, DM, HF, SxSI, TIMI < 3 flow, hemoglobin, troponin, and ejection fraction; (2) base + GRACE model incorporating the GRACE score; and (3) base + GRACE + IPI model, adding the inflammatory-prognostic index. All models identified male gender, presence of diabetes, presence of HF, lower LVEF, higher SxSI, higher baseline TnI levels, higher GRACE score, and IPI score as independent predictors of MACCEs (Table 4).

A risk reclassification analysis demonstrated progressive improvement across models. The base + GRACE model showed a modest reclassification benefit with an NRI of 0.009 (95% CI: −0.006 to 0.025, *p* = 0.232), although this did not reach statistical significance. However, it demonstrated significant discrimination improvement with an IDI of 0.041 (95% CI: 0.034–0.048, *p* < 0.001), corresponding to a 20.0% relative improvement in discrimination slope. The base + GRACE + IPI model achieved statistically significant reclassification improvement, with an NRI of 0.010 (95% CI: 0.004–0.016, *p* = 0.001) and an IDI of 0.044 (95% CI: 0.038–0.051, *p* < 0.001), representing a 21.7% relative improvement in discrimination. The progressive improvement from base + GRACE (borderline significant) to base + GRACE + IPI (highly significant) suggests that the combination of inflammatory biomarkers provides substantial enhancement to cardiovascular risk prediction beyond traditional risk factors alone.

**Figure 2:**
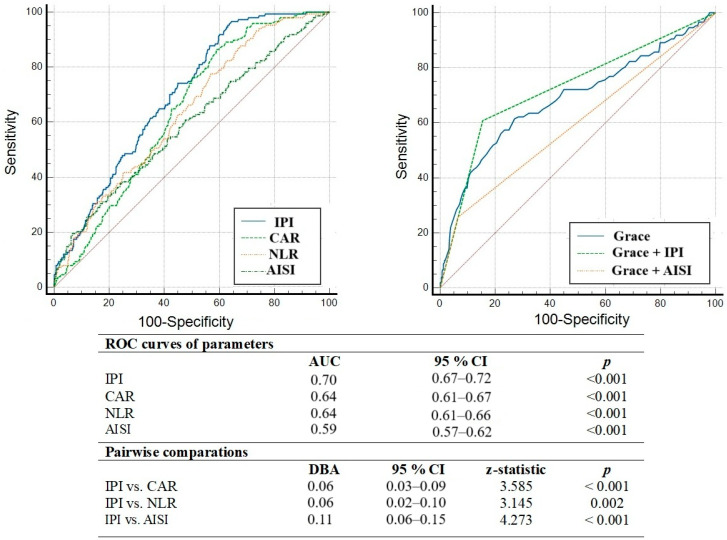
Comparison of discriminatory abilities of the IPI, CAR, NLR, and AISI, determined by the DeLong test, in determining the development of one-year MACCEs. Abbreviations: ROC, the receiving operating characteristic; AUC, the area under the curve; CI, confidence interval; IPI, inflammatory prognostic index; CAR, C-reactive protein-to-albumin ratio; NLR, neutrophil-to-lymphocyte ratio; AISI, aggregate index of systemic inflammation; DBA, difference between areas; MACCEs, major adverse cardiovascular and cerebrovascular events.

We also performed an ROC curve analysis to test whether the IPI, AISI, CAR, and the NLR had sufficient predictive performance and discrimination abilities. The ROC analysis revealed that only the discriminating power of the IPI for one-year MACCEs (AUC: 0.70, 95% CI = 0.67–0.72, *p* < 0.001) was adequate. Furthermore, the IPI was found to be superior to other inflammatory indices, such as the CAR, NLR, and AISI, in a comparative analysis of ROC curves (Figure 2). In addition, the cut-off value for the IPI was above 2.43, which had 95% sensitivity and 37% specificity for MACCEs.

**Figure 3:**
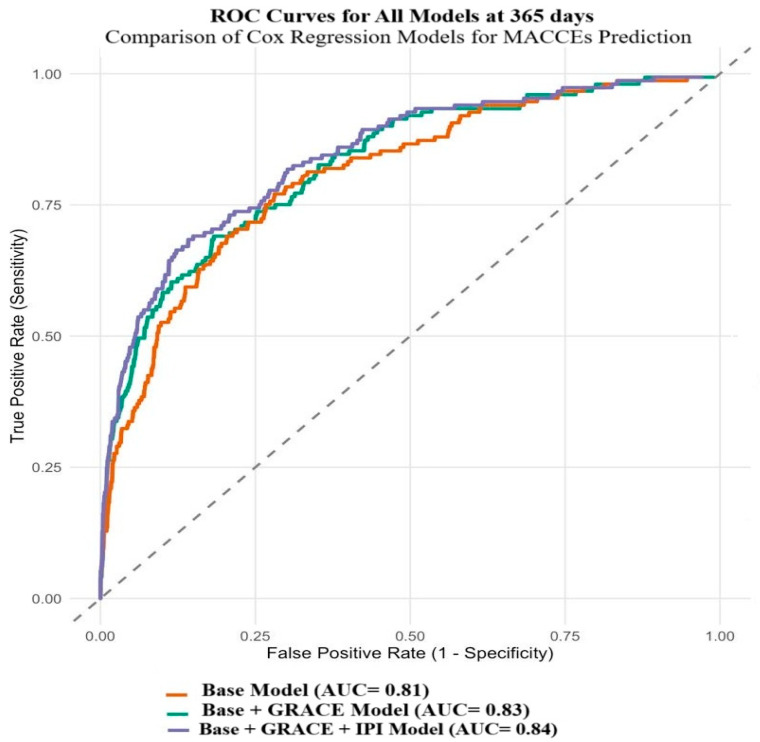
Comparison of the discriminatory abilities of models determined by DeLong’s test in determining the development of one-year MACCEs. Abbreviations: ROC, the receiving operating characteristic; MACCEs, major adverse cardiovascular and cerebrovascular events; AUC, the area under the curve; GRACE, Global Registry of Acute Coronary Events; IPI, inflammatory prognostic index.

Furthermore, the base model incorporating traditional cardiovascular risk factors demonstrated good discriminative ability with a C-index of 0.81 (95% CI: 0.79–0.83) and explained 18.2% of the outcome variance (Nagelkerke R^2^ = 0.182). The addition of GRACE to the base model enhanced discrimination to 0.83 (95% CI: 0.81–0.85) with increased explanatory power (R^2^ = 0.211), representing a meaningful improvement over the base model, although the discriminative enhancement did not reach statistical significance (DeLong test *p* = 0.067). Further enhancement was achieved with the inclusion of the IPI alongside GRACE, yielding the highest discriminative performance with a C-index of 0.84 (95% CI: 0.82–0.86) and explanatory power of 23.2% (R^2^ = 0.232), with statistically significant improvement in discrimination compared to the base model (DeLong test *p* = 0.001; Figure 3).

**Figure 4:**
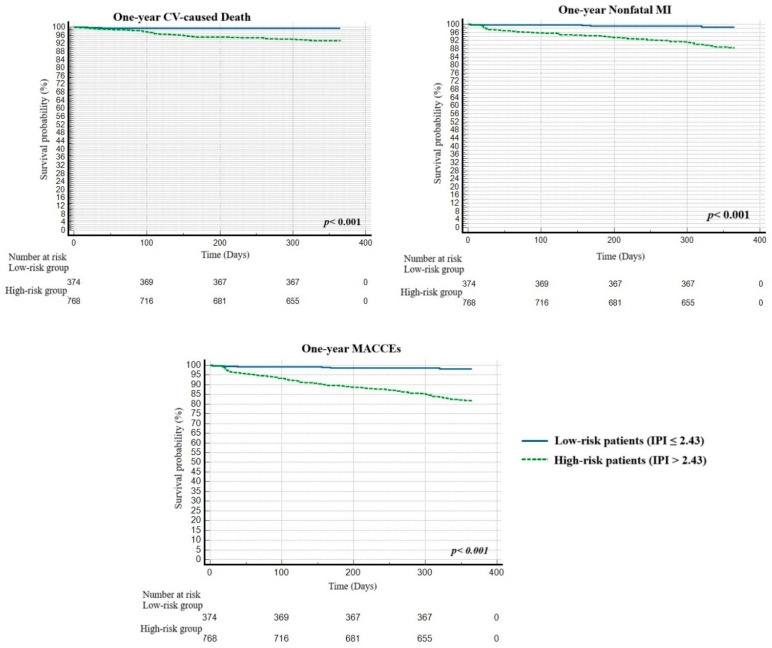
The Kaplan–Meier plots of survival curves of patients with high (green line) and low (blue line) IPI categories. Abbreviations: CV, cardiovascular; MI, myocardial infarction; MACCEs, major adverse cardiovascular and cerebrovascular events; IPI, inflammatory prognostic index.

Moreover, Kaplan–Meier curves revealed that high-risk patients with an IPI value greater than 2.43 had significantly more adverse events than the low-risk group during the follow-up period (*p* < 0.001) (Figure 4).

## 4. Discussion

The main findings of the current investigation are as follows: (1) Male gender, presence of diabetes, presence of HF, lower LVEF, higher SxSI, higher GRS, higher baseline TnI levels, and higher preprocedural IPI values were found to be independent predictors for one-year MACCEs. (2) Among inflammation-based scores, including the IPI, AISI, CAR, and the NLR, only the IPI had adequate discriminatory power; it was also superior to the other aforementioned inflammation indices. (3) A preprocedural IPI value > 2.43 exhibited discrimination ability for MACCEs with 95% sensitivity and 37% specificity. (4) Adding the IPI to the baseline model significantly improved the discrimination ability in assessing the likelihood of MACCEs occurring within a year. (5) High-risk individuals experienced a statistically higher frequency of adverse events during follow-up when compared with low-risk individuals.

Inflammation plays a central role in both initiating and advancing atherosclerosis through various mediators, triggering vascular inflammation, disrupting hemostasis, causing plaque rupture, and ultimately leading to acute coronary thrombosis [9,10]. Moreover, there are data from the literature suggesting that inflammation severity is the primary mechanism linked to clinical instability in patients with NSTEMI [10]. Indeed, previous investigations examining inflammation’s prognostic significance in ACS patients have demonstrated that various inflammatory markers, such as the CAR, NLR, and AISI, show potential for enhancing risk assessment, predicting patient outcomes, and improving overall clinical results [1,7,12,13,14,15,32]. Recently, Menekse and colleagues demonstrated that the CAR was related to in-hospital mortality in a total of 308 NSTEMI patients undergoing PCI [13]. In a meta-analysis conducted by Banahene et al., including 37 randomized and non-randomized trials reporting the effect of NLR values in adult patients with MI, including STEMI and NSTEMI, one of these markers, the NLR, emerged as a predictor of adverse events and overall mortality in individuals with ACS treated with PCI [1]. Furthermore, Jiang et al. recently suggested that a high AISI level, another inflammatory marker derived from complete blood count components, was associated with an increased risk of in-hospital mortality in a total of 1044 patients presenting with acute myocardial infarction (STEMI and NSTEMI), and thus may be an early marker of adverse prognosis [15].

The IPI, a recently described nutritional/inflammatory marker, includes the NLR as well as the CAR derived from serum albumin and serum CRP. It has also been postulated that compared to the NLR and the CAR individually, the IPI may demonstrate enhanced predictive capability [14]. Although initially developed to assess prognosis in various cancer patients, the predictive role of the IPI in various CVDs has been investigated in only a few recent studies [14,16,33,34,35]. According to the findings of Jiang et al., the IPI was strongly correlated with clinical outcomes and independently predicted the risk of contrast-induced nephropathy and adverse postprocedural outcomes, including severe arrhythmia and MI [16]. Saylık et al. have also reported the significant association between higher baseline IPI levels and the no-reflow phenomenon in STEMI patients who undergo primary PCI [14]. Another study conducted by Yang et al. demonstrated a significant association between increased IPI levels and six-month mortality in individuals with heart failure. They also showed that it has a better predictive value than other inflammation-based markers consisting of only WBC elements, such as the systemic immune inflammation index and systemic inflammatory response index [35]. Additionally, to the best of our knowledge, no existing research has addressed evaluating the prognostic value of the IPI in NSTEMI patients undergoing PCI. Moreover, the relationship between the IPI and MACCEs maintained its significance even after we controlled for various confounding factors through both univariate and multivariate logistic regression analyses. Moreover, although previous studies have shown that the CAR and AISI are associated with in-hospital mortality, there is no study in the literature demonstrating the association of these aforementioned parameters with mid- to long-term adverse events in the NSTEMI population. Thus, in addition to being the first study to demonstrate the prognostic significance of the IPI, another point to consider is that it demonstrates that the AISI and the CAR are associated with one-year MACCEs. In addition, it should also be kept in mind that the IPI was found to have better predictive performance than other inflammatory indices, including the CAR, NLR, and AISI.

It is known that NSTEMI patients generally have better short-term outcomes than STEMI patients, but despite medical and clinical advances, their long-term prognosis remains poor [3,4,5]. Therefore, it is extremely important to identify risk factors that may contribute to risk stratification, thereby improving the long-term and, therefore, overall prognosis in NSTEMI patients. Due to its robust validation and widespread acceptance, the GRACE risk score remains the preferred method for risk stratification of ACS patients in international recommendations [23,24,25]. The GRACE score is based primarily on clinical, electrocardiographic data, and cardiac damage markers and does not include inflammatory markers. However, since there has been evidence in recent years that inflammatory markers are valuable for evaluating prognosis in heart diseases, emerging data suggest that adding these markers to risk scores may strengthen their prognostic utility [10,32]. In support of this thesis, the current study has demonstrated that combining the IPI with the GRACE score results in markedly improved predictive performance and discriminatory capacity compared to the GRACE score alone. In this regard, it is also noteworthy that this study demonstrates that the IPI may provide a significant improvement in one-year cardiovascular risk prediction in NSTEMI patients beyond traditional risk factors, guiding more targeted preventive interventions aimed at minimizing adverse outcomes.

These findings may be due to the fact that inflammation and malnutrition are closely related to the development of atherosclerosis and CVD. The association linking these three components has been increasingly recognized in the recent literature, as malnutrition-inflammatory atherosclerosis syndrome has been associated with increased mortality [36]. Inflammation plays an important role in the progression of atherosclerosis by impairing cardiovascular endothelial function, mobilizing leukocytes, and inducing the expression of proinflammatory cytokines such as elastase, myeloperoxidase, and free oxygen radicals that contribute to oxidative stress, ischemic myocardial damage, and ischemia-reperfusion injury [37,38,39]. It has also been implicated in the promotion of anorexia, increased tissue catabolism, and a decreased synthesis rate of albumin, which together may drive the malnutrition, muscle decline, and weight loss characteristic of frailty [37,40]. Additionally, pro-inflammatory cytokines such as IL-6 released from inflammatory cells cause anorexia, decreased dietary protein intake, and further hypercatabolism [40,41]. Furthermore, among patients with ACS, malnutrition is recognized as a factor linked to the progression of atherosclerosis and poorer clinical outcomes, including higher mortality and major adverse cardiac events [37]. In clinical practice, serum albumin is frequently used to identify malnutrition [13,37]. Hypoalbuminemia is associated with decreased antioxidant, anti-inflammatory, and antiplatelet aggregation activity, leading to impaired endothelial function, increased blood viscosity, and oxidative stress, which are associated with increased risk of cardiovascular mortality [38,42]. Moreover, some studies suggest that albumin may be more reliable than CBC parameters, given its ability to reflect both nutritional health and systemic inflammation [7]. Also, in addition to its important role in the progression of atherosclerosis, CRP, as a positive acute-phase reactant, is also an indicator of the inflammatory status that contributes to the aggravation of malnutrition [43]. The IPI is a composite index derived from albumin, CRP, and WBC, which are indicators related to nutrition and inflammation, respectively. In this context, the above-mentioned could be several possible pathophysiological mechanisms that explain both the predictive role of the IPI, composed of CRP, albumin, and the NLR, and its ability to outperform other scores derived from WBC counts alone.

Consistent with published data, prior heart failure, male gender, diabetes, higher initial troponin concentrations, elevated SxSI, and impaired LVEF emerged as independent risk factors for MACCEs in our cohort [32,37,44,45].

There are various limitations of our study that must be taken into consideration. An initial limitation is that this study, based exclusively on hospital information system data, was conducted at a single center and involved a relatively modest sample size, which may reduce external validity while increasing internal consistency in clinical practice and laboratory methods. In addition, the retrospective nature of the study carries an inherent risk of selection bias and limits the ability to draw causal inferences. Second, by focusing exclusively on admission IPI measurements, their dynamic fluctuations over time have been ignored. Thus, longitudinal studies are also needed to better evaluate the impact of these variables on prognosis. Third, although we made extensive efforts to adjust for key covariates, the potential influence of unmeasured factors, such as issues with treatment adherence, cannot be excluded and may have introduced bias. A fourth limitation is the lack of investigation into additional confounding factors, such as unrecognized cancer, mental health disorders, or hypothyroidism, which could also contribute to malnutrition. Fifth, because the SxSI score could not be calculated, patients who underwent CABG were excluded from the study. Furthermore, only NSTEMI patients treated with PCI were included, limiting the generalizability of the findings to the broader NSTEMI population, comprising individuals who were decided to undergo medical treatment or CABG. Sixth, the retrospective and single-center nature of the current study may also affect the accuracy and generalizability of the findings. Thus, the findings obtained in our study are exploratory in nature and should be evaluated as hypothesis-generating rather than being generalized. Therefore, we emphasize that better-designed, prospective, multicenter further studies with larger populations are needed to confirm and expand the results of our study. Despite these limitations, this study introduces a novel and easily obtainable nutritional-inflammatory marker that may contribute to the risk stratification in NSTEMI patients undergoing PCI.

## 5. Conclusions

In conclusion, this study highlights the potential value of the IPI in predicting one-year MACCEs among NSTEMI patients undergoing PCI. Our findings demonstrate that the IPI offers better discriminatory ability, predictive performance, and risk stratification benefits in this patient population. Therefore, the superiority of the IPI over other indices such as the CAR, NLR, and AISI may be related to the comprehensive assessment of both inflammation and nutritional status, providing a more holistic assessment of the patient’s condition. Additionally, combining baseline IPI levels with the GRACE score resulted in improved predictive accuracy for one-year MACCEs compared to the GRACE score alone. Given its simplicity, accessibility, and cost-effectiveness, the IPI may serve as a practical tool for clinicians to identify high-risk patients who require a more aggressive treatment approach and closer clinical follow-up, thereby individualizing treatment strategies. However, larger prospective studies are still needed to assess the accuracy of the IPI in predicting adverse cardiovascular outcomes in the setting of NSTEMI and to better define its role in clinical decision-making.

## Figures and Tables

**Table 1:** Demographic and clinical parameters of individuals with low and high IPI according to median value.

Variables	All Population (*n* = 1142)	Low IPI (<3.40) (*n* = 957; 83.8%)	High IPI (≥3.40) (*n* = 185; 16.2%)	*p*
Male gender, *n*%	848 (74.3)	738 (77.1)	110 (59.5)	<0.001
Age, years	61.9 ± 12.5	60.3 ± 12.4	70.3 ± 9.8	<0.001
BMI (kg/m^2^)	27.7 ± 3.3	27.7 ± 3.3	27.5 ± 3.1	0.482
Hypertension, *n* (%)	659 (57.7)	531 (55.5)	128 (69.2)	0.001
Diabetes, *n* (%)	402 (35.2)	312 (32.6)	90 (48.6)	<0.001
Hyperlipidemia, *n* (%)	532 (46.6)	454 (47.4)	78 (42.2)	0.188
Smoking, *n* (%)	504 (44.1)	431 (45.0)	73 (39.5)	0.162
Family history, *n* (%)	407 (35.6)	338 (35.3)	69 (37.3)	0.607
CAD history, *n* (%)	483 (42.3)	394 (41.2)	89 (48.1)	0.080
Previous MI, *n* (%)	341 (29.9)	277 (28.9)	64 (34.6)	0.124
Previous PCI, *n* (%)	360 (31.5)	302 (31.6)	58 (31.4)	0.956
PAD history, *n* (%)	32 (2.8)	24 (2.5)	8 (4.3)	0.171
Heart Failure, *n* (%)	208 (18.2)	152 (15.9)	56 (30.3)	<0.001
CRF, *n* (%)	144 (12.6)	94 (9.8)	50 (27.0)	<0.001
Dialysis, *n* (%)	13 (1.1)	8 (0.8)	5 (2.7)	0.028
Killip III-IV, *n* (%)	97 (8.5)	54 (5.6)	43 (23.2)	<0.001
Hemodynamic instability, *n* (%)	31 (2.7)	18 (1.9)	13 (7.0)	<0.001
GRACE risk score	98.1 ± 22.9	92.8 ± 20.3	125.6 ± 14.5	<0.001
LVEF, %	51.3 ± 9.8	51.5 ± 9.7	50.3 ± 10.6	0.134
Medications, *n* (%)				
Acetylsalicylic acid	413 (36.2)	340 (35.5)	73 (39.5)	0.308
ADP blockers	132 (11.6)	110 (11.5)	22 (11.9)	0.877
OACs, *n* (%)	56 (4.9)	36 (3.8)	20 (10.8)	<0.001
Beta-blockers	335 (29.3)	274 (29.3)	61 (33.0)	0.235
RAS blockers	490 (42.9)	400 (41.8)	90 (48.6)	0.085
CCBs, *n* (%)	421 (36.9)	341 (35.6)	80 (43.2)	0.049
Statin	250 (21.9)	204 (21.3)	46 (24.9)	0.285
Antianginals, *n* (%)	110 (9.6)	96 (10.0)	14 (7.6)	0.298
OADs, *n* (%)	382 (33.5)	296 (30.9)	86 (46.5)	<0.001
Insulin, (%)	67 (5.9)	47 (4.9)	20 (10.8)	0.002
Syntax score I	20.1 ± 6.0	19.9 ± 5.8	20.8 ± 7.0	0.070
TIMI < 3 flow, *n* (%)	100 (8.8)	82 (8.6)	18 (9.7)	0.609
TVR, year, *n* (%)	37 (3.2)	26 (2.7)	11 (5.9)	0.023
Nonfatal MI, 30 days, *n* (%)	23 (2.0)	12 (1.3)	11 (5.9)	<0.001
Nonfatal stroke, 30 days, *n* (%)	2 (0.2)	1 (0.1)	1 (0.5)	0.194
CV-caused mortality, 30 days, *n* (%)	5 (0.4)	2 (0.2)	3 (1.6)	0.008
MACCEs, 30 days, *n* (%)	30 (2.6)	15 (1.6)	15 (8.1)	<0.001
Nonfatal MI, year, *n* (%)	90 (7.9)	45 (4.7)	45 (24.3)	<0.001
Nonfatal stroke, year, *n* (%)	7 (0.6)	5.0 (0.5)	2 (1.1)	0.373
CV-caused mortality, year, *n* (%)	51 (4.5)	23 (2.4)	28 (15.1)	<0.001
MACCEs, 1 year, *n* (%)	148 (13.0)	73 (7.6)	75 (40.5)	<0.001

Abbreviations: BMI, body mass index; CAD, coronary artery disease; MI, myocardial infarction; PCI, percutaneous coronary intervention; PAD, peripheral arterial disease; CRF, chronic renal failure; GRACE, the Global Registry of Acute Coronary Events; LVEF, left ventricular ejection fraction; ADP, adenosine diphosphate; OACs, oral anticoagulants; RAS, the renin-angiotensin system; CCBs, calcium channel blockers; OADs, oral antidiabetic drugs; TIMI, thrombolysis in myocardial infarction; CR, complete revascularization during the hospitalization; TVR, target vessel revascularization; CV, cardiovascular; MACCEs, major cardiovascular and cerebrovascular events.

**Table 2:** Laboratory parameters of the study cohort grouped according to the median IPI value.

Variables	All Population (*n* = 1142)	Low IPI (<3.40) (*n* = 957; 83.8%)	High IPI (≥3.40) (*n* = 185; 16.2%)	*p*
Glucose, mg/dL	112.9 ± 27.2	112.4 ± 26.6	115.5 ± 30.2	0.151
eGFR, mL/min/1.73 m^2^	81.0 ± 23.7	83.8 ± 22.4	66.5 ± 24.6	<0.001
Uric acid, mg/dL	5.49 ± 1.7	5.52 ± 1.7	5.38 ± 1.6	0.329
Albumin, g/dL	3.88 ± 0.48	3.91 ± 0.47	3.73 ± 0.50	<0.001
CRP, mg/dL, IQR	5.36 [3.45–8.20]	4.95 [3.20–7.68]	7.76 [5.55–9.80]	<0.001
Troponin I, ng/mL, IQR	0.06 [0.02–0.21]	0.06 [0.02–0.19]	0.07 [0.03–0.30]	0.047
CAR, IQR	1.42 [0.88–2.15]	1.28 [0.82–1.99]	2.0 [1.49–2.74]	<0.001
TC, mg/dL	193.5 ± 43.7	194.2 ± 44.2	189.5 ± 40.4	0.174
LDL-C, mg/dL	117.4 ± 37.7	118.2 ± 37.8	113.2 ± 37.4	0.10
HDL-C, mg/dL	39.2 ± 10.3	39.2 ± 10.2	39.53 ± 10.5	0.907
Triglycerides, mg/dL, IQR	122 [99.0–167.0]	122 [99.0–164]	122 [99.0–179.5]	0.112
Hemoglobin, g/dL	13.5 ± 1.9	13.7 ± 1.8	12.6 ± 1.9	<0.001
WBC, 10^9^/L	8.51 ± 2.2	8.47 ± 2.2	8.77 ± 2.6	<0.001
Neutrophils, 10^9^/L	5.22 ± 1.8	5.13 ± 1.8	5.76 ± 2.0	<0.001
Lymphocytes, 10^9^/L, IQR	2.06 [1.60–2.59]	2.13 [1.68–2.65]	1.74 [1.28–2.36]	<0.001
Monocytes, 10^9^/L, IQR	0.53 [0.43–0.66]	0.53 [0.43–0.65]	0.57 [0.45–0.71]	0.037
Platelets, 10^9^/L	254.1 ± 74.3	253.0 ± 72.1	259.9 ± 84.8	0.243
NLR, IQR	2.36 [1.84–3.24]	2.29 [1.78–3.06]	3.05 [2.33–4.22]	<0.001
AISI, IQR	307.8 [206.6–506.8]	289.9 [194.0–470.5]	429.9 [267.8–733.7]	<0.001
IPI, IQR	3.40 [1.94–5.85]	2.99 [1.71–5.14]	6.08 [4.50–8.28]	<0.001

Abbreviations: eGFR, estimated glomerular filtration rate; CRP, C-reactive protein; CAR, C-reactive protein-to-albumin ratio; TC, total cholesterol; LDL-C, low-density lipoprotein cholesterol; HDL-C, high-density lipoprotein cholesterol; WBC, white blood count; NLR, neutrophil-to-lymphocyte ratio; AISI, aggregate index of systemic inflammation; IPI, inflammatory prognostic index.

**Table 3:** Univariate Cox regression analyses of all parameters associated with MACCEs.

Variables	HR (95% CI)	*p*	Variables	HR (95% CI)	*p*
Male gender	1.83 (1.31–2.55)	<0.001	TIMI < 3 flow	1.83 (1.15–2.90)	0.010
Age (years)	1.03 (1.02–1.05)	<0.001	Troponin I (ng/mL)	1.44 (1.35–1.54)	<0.001
Diabetes	2.24 (1.62–3.09)	<0.001	CRP (mg/dL)	1.08 (1.04–1.13)	<0.001
Previous CAD	1.49 (1.08–2.05)	0.016	Albumin (g/dL)	0.47 (0.35–0.63)	<0.001
Previous HF	2.95 (2.11–4.12)	<0.001	Hemoglobin (g/dL)	0.81 (0.75–0.88)	<0.001
Previous CRF	3.07 (2.15–4.39)	<0.001	Lymphopenia	0.52 (0.41–0.65)	<0.001
Killip class II-IV	1.66 (1.02–2.68)	0.040	CAR	1.41 (1.22–1.62)	<0.001
LVEF (%)	0.94 (0.93–0.96)	<0.001	NLR	1.22 (1.14–1.30)	<0.001
GRACE risk score	1.03 (1.02–1.04)	<0.001	AISI	1.00 (1.00–1.00)	<0.001
Syntax score I	1.06 (1.03–1.08)	<0.001	IPI	1.09 (1.07–1.11)	<0.001

Abbreviations: MACCEs, major adverse cardiovascular and cerebrovascular events; HR, hazard ratio; CI, confidence interval; CAD, coronary artery disease; HF, heart failure; CRF, chronic renal failure; LVEF (%), left ventricular ejection fraction; GRACE, Global Registry of Acute Coronary Events; TIMI, thrombolysis in myocardial infarction; IPI, inflammatory prognostic index.

**Table 4:** Factors that were found to be independently associated with the MACCEs in multivariate Cox regression analysis.

Variables	Base Model	*p*	Base + GRACE Model	*p*	Base + GRACE + IPI Model	*p*
	HR (95% CI)		HR (95% CI)		HR (95% CI)	
Male gender	1.49 (1.05–2.12)	0.027	1.47 (1.02–2.09)	0.036	1.53 (1.07–2.21)	0.021
Diabetes	1.82 (1.29–2.55)	<0.001	1.43 (1.00–2.03)	0.048	1.62 (1.13–2.31)	0.008
Previous CAD	1.08 (0.78–1.50)	0.641	1.10 (0.79–1.53)	0.577	1.09 (0.78–1.51)	0.620
Previous HF	2.11 (1.50–2.97)	<0.001	1.66 (1.16–2.37)	0.005	1.70 (1.19–2.42)	0.003
LVEF (%)	0.95 (0.93–0.96)	<0.001	0.94 (0.93–0.95)	<0.001	0.94 (0.93–0.96)	<0.001
Syntax score I	1.04 (1.02–1.07)	<0.001	1.04 (1.01–1.06)	0.004	1.03 (1.00–1.05)	0.019
TIMI < 3 flow	1.17 (0.72–1.91)	0.519	1.11 (0.68–1.82)	0.668	1.24 (0.76–2.03)	0.383
Troponin I (ng/mL)	1.39 (1.29–1.50)	<0.001	1.41 (1.30–1.53)	<0.001	1.43 (1.32–1.55)	<0.001
Hemoglobin (g/dL)	0.90 (0.82–0.98)	0.021	0.97 (0.88–1.08)	0.618	0.98 (0.89–1.09)	0.734
GRACE risk score	-	-	1.02 (1.01–1.03)	<0.001	1.02 (1.01–1.03)	<0.001
IPI	-	-	-	-	1.07 (1.04–1.09)	<0.001

Abbreviations: MACCEs, major adverse cardiovascular and cerebrovascular events; HR, hazard ratio; CI, confidence interval; CAD, coronary artery disease; HF, heart failure; LVEF (%), left ventricular ejection fraction; TIMI, thrombolysis in myocardial infarction; GRACE, Global Registry of Acute Coronary Events; IPI, inflammatory prognostic index.

## Data Availability

All data generated or analyzed during this study are included in the published article. Additional details can be obtained by contacting the corresponding author.
